# Supplementary material for: Identifying Genetic Mutation Status in Patients with Colorectal Cancer Liver Metastases Using Radiomics-Based Machine-Learning Models
Source: Cancers (Basel). 2023 Nov 29;15(23):5648. doi: 10.3390/cancers15235648 (PMC10705206; doi:10.3390/cancers15235648)
Supplement: Supplementary file 1 [file cancers-15-05648-s001.zip › cancers-2648724-supplementary.pdf]

SUPPLEMENT (S1-S5)

S1. CT acquisition parameters

	Development cohort (CAIRO5)
Slice thickness (mm)	5.0 [3.0 – 5.0]
Pixel spacing (mm)	0.73 [0.68 – 0.78]
Tube voltage (kvP)	120 [100 – 120]
Total collimation width (mm)	40 [40 – 40]
Exposure (mAs)	50 – 293
CT dose index (mGy)	2 – 15

Note – Values are displayed as median with [interquartile range] or as range.

	External validation cohort (NKI)
Slice thickness (mm)	1.0 [1.0 – 1.5]
Pixel spacing (mm)	0.79 [0.76 – 0.85]
Tube voltage (kvP)	120 [120 – 120]
Total collimation width (mm)	19 [19 – 29]
Exposure (mAs)	25 - 299
CT dose index (mGy)	2 – 33

Note – Values are displayed as median with [interquartile range] or as range.

S2. Radiomics Quality Score

Criteria		Points	Study
1	Image protocol quality - well-documented image protocols (for example, contrast, slice thickness, energy, etc.) and/or usage of public image protocols allow reproducibility/replicability	+ 1 (if protocols are well-documented) + 1 (if public protocol is used)	1
2	Multiple segmentations - possible actions are: segmentation by different physicians/algorithms/software, perturbing segmentations by (random) noise, segmentation at different breathing cycles. Analyse feature robustness to segmentation variabilities	+ 1	-
3	Phantom study on all scanners - detect inter-scanner differences and vendor-dependent features. Analyse feature robustness to these sources of variability	+ 1	-
4	Imaging at multiple time points - collect images of individuals at additional time points. Analyse feature robustness to temporal variabilities (for example, organ movement, organ expansion/ shrinkage)	+ 1	-
5	Feature reduction or adjustment for multiple testing - decreases the risk of overfitting. Overfitting is inevitable if the number of features exceeds the number of samples. Consider feature robustness when selecting features	- 3 (if neither measure is implemented) + 3 (if either measure is implemented)	3
6	Multivariable analysis with non radiomics features (for example, EGFR mutation) - is expected to provide a more holistic model. Permits correlating/inferencing between radiomics and non radiomics features	+ 1	1
7	Detect and discuss biological correlates - demonstration of phenotypic differences (possibly associated with underlying gene–protein expression patterns) deepens understanding of radiomics and biology	+ 1	1
8	Cut-off analyses - determine risk groups by either the median, a previously published cut-off or report a continuous risk variable. Reduces the risk of reporting overly optimistic results	+ 1	-
9	Discrimination statistics - report discrimination statistics (for example, C-statistic, ROC curve, AUC) and their statistical significance (for example, p-values, confidence intervals). One can also apply resampling method (for example, bootstrapping, cross-validation)	+ 1 (if a discrimination statistic and its statistical significance are reported) + 1 (if a resampling method technique is also applied)	2
10	Calibration statistics - report calibration statistics (for example, Calibration-in-the-large/slope, calibration plots) and their statistical significance (for example, *P*-values, confidence intervals). One can also apply resampling method (for example, bootstrapping, cross-validation)	+ 1 (if a calibration statistic and its statistical significance are reported) + 1 (if a resampling method technique is also applied)	2
11	Prospective study registered in a trial database - provides the highest level of evidence supporting the clinical validity and usefulness of the radiomics biomarker	+ 7 (for prospective validation of a radiomics signature in an appropriate trial)	-
12	Validation - the validation is performed without retraining and without adaptation of the cut-off value, provides crucial information with regard to credible clinical performance	- 5 (if validation is missing) + 2 (if validation is based on a dataset from the same institute) + 3 (if validation is based on a dataset from another institute) + 4 (if validation is based on two datasets from two distinct institutes) + 4 (if the study validates a previously published signature) + 5 (if validation is based on three or more datasets from distinct institutes)<br>*Datasets should be of comparable size and should have at least 10 events per model feature	3
13	Comparison to 'gold standard' - assess the extent to which the model agrees with/is superior to the current 'gold standard' method (for example, TNM-staging for survival prediction). This comparison shows the added value of radiomics	+ 2	2
14	Potential clinical utility - report on the current and potential application of the model in a clinical setting (for example, decision curve analysis).	+ 2	2
15	Cost-effectiveness analysis - report on the cost-effectiveness of the clinical application (for example, QALYs generated)	+ 1	-
16	Open science and data - make code and data publicly available. Open science facilitates knowledge transfer and reproducibility of the study	+ 1 (if scans are open source) + 1 (if region of interest segmentations are open source) + 1 (if code is open source) + 1 (if radiomics features are calculated on a set of representative ROIs and the calculated features and representative ROIs are open source)	-
Total points (36 = 100%)			17 (47%)

S3. Pyradiomics feature extraction settings

The Pyradiomics feature extraction process requires several settings to be determined based on the imaging data at hand. According to the Pyradiomics documentation, the ideal number of bins lies between 16-128 bins. Their suggestion in determining the best bin width, is extracting the range feature from the dataset and choosing a bin width such that the range/bin width ratio remains within the range of 16-128. [28] Using this method, a bin width of 10 was chosen for the dataset in this study. The voxel array shift describes a value that is added to the gray level intensity value for certain features to prevent negative values. Since CT scans have a minimum Hounsfield unit (HU) value of -1000, this parameter was set at 1000 to prevent negative numbers. In addition, to ensure isotropic voxel spacing with equal distances between neighbouring voxels, the images were interpolated using the sitKBSpline interpolator. Lastly, all possible feature groups were extracted from both the original and the wavelet filtered VOIs. All features were standardized using z-score normalization in advance. The feature extraction settings are summarized in Table S3.

Table S1.

Bin width	10
Voxel array shift	1000
Resampled pixel spacing	1 x 1 x 1 mm3
Interpolator	sitkBSpline
Filter	Wavelet
Feature groups	First order, shape, glcm, glrlm, glszm, gldm, ngtdm

S4. Model configurations

Table S2 – Model parameters for Random Forest classifier

Number of trees	25
Tree splitting options	Information gain ratio
Minimum leaf size	5
Maximum depth	20
Number of inputs to consider per split	8

Table S3 - Model parameters for Gradient Boosting classifier

Number of trees	257
Learning rate	0.2
Subsampling rate	0.5
L1 regularization	0.1
L2 regularization	1
Minimum leaf size	5
Maximum depth	4

Table S4 - Model parameters for Ensemble classifier

Posterior probabilities	Voting
Voting posterior probabilities	Proportion

S5. Selected features

From the 851 radiomics features using the Pyradiomics package (v3.0.1) [28], the following 10 features were selected by the random forest features selection to be used as input for the classification models:

- Shape: Least Axis Length (original feature)
- Gray Level Dependence Matrix: Dependence Entropy (original feature)
- Gray Level Co-occurrence Matrix: Inverse Difference Moment Normalized (HHH wavelet filter feature)
- Gray Level Co-occurrence Matrix: Maximal Correlation Coefficient (HHH wavelet filter feature)
- Neighbouring Gray Tone Difference Matrix: Busyness (HLH wavelet filter feature)
- Gray Level Size Zone Matrix: Zone Entropy (HLL wavelet filter feature)
- Gray Level Co-occurrence Matrix: Correlation (LLH wavelet filter feature)
- Gray Level Co-occurrence Matrix: Cluster Shade (LLL wavelet filter feature)
- Gray Level Co-occurrence Matrix: Informational Measure of Correlation 2 (LLL wavelet filter feature)
- Gray Level Run Length Matrix: Run Entropy (LLL wavelet filter feature)

A description of the definition of each feature can be found in the Pyradiomics documentation[28].
